# Rationale and methodology of an ongoing Maltese longitudinal cohort study focusing on obesity: a decade of population health surveillance

**DOI:** 10.1016/j.gloepi.2026.100298

**Published:** 2026-11-04

**Authors:** Elizabeth Grech, Neville Calleja, Keith Calleja, Axel Tonna, Grazia Magro, Sarah Cuschieri

**Affiliations:** University of Malta, Faculty of Medicine and Surgery, Triq Dun Karm, Msida MSD 2080, Malta

**Keywords:** Obesity, Preventive medicine, Public health, Epidemiology

## Abstract

**Background/objective:**

Obesity prevalence in Malta is among the highest in Europe and continues to rise. Population metabolic health is dynamic and longitudinal evidence is required to understand temporal changes in adiposity and their determinants within the Maltese population. This study aims to investigate 10-year longitudinal changes in adiposity and examine associations between socio-demographic, socio-economic, lifestyle, clinical, and biological factors and overweight/obesity outcomes among adults in Malta.

**Methods:**

SAHHTEK 2.0 is a 10-year longitudinal follow-up of the 2016 SAHHTEK cohort. Of 1861 original participants, 455 were systematically selected for invitation to reassessment in 2026. Questionnaire, anthropometric, blood pressure, fasting glucose, and lipid measurements will be repeated. Prespecified multivariable regression models will examine longitudinal associations between baseline exposures and adiposity outcomes in 2026, with BMI as the primary outcome and adjustment for baseline adiposity and prespecified covariates.

**Results:**

At the time of writing, 268 of the targeted 455 participants have been recruited. Data collection is ongoing, and analyses will quantify changes in adiposity over a 10-year period and identify key determinants of overweight and obesity.

**Conclusions:**

Findings of this study will provide essential evidence on obesity trajectories and modifiable risk factors, supporting targeted prevention strategies and informing national public health policy.

## Introduction

The prevalences of both overweight and obesity in the Maltese Islands have risen progressively over the years [1]. The last representative national cross-sectional study carried out in Malta was titled ‘*SAHHTEK - The University of Malta Health and Wellbeing study’* and was conducted between 2014 and 2016 [2]. This study explored the prevalence rates of type 2 diabetes mellitus, impaired fasting blood glucose (IFG) and their co-determinants including overweight-obesity status, hypertension, and metabolic syndrome at a population level. The study provided evidence-based data and explored links between various socio-demographic, lifestyle, and biological factors [3]. The metabolic status of a population is not static and fluctuations occur over time as a result of various factors such as age, a changing environment, socio-demographics and other cultural and social factors [4].

The COVID-19 pandemic is a factor that is expected to have influenced the health status of the population [5]. Direct links between the body's metabolic and endocrine systems and COVID-19 have been established, leaving room for further research pertaining to clinical challenges in dealing with new phenomena such as Long COVID [6]. Recent studies have explored various disease trajectories before, during and after the COVID pandemic [7], [8]. The application of the before-after pandemic framework provides comparative insights into pandemic-related behavioural changes.

Establishing population-specific risk factors for obesity would enable tailored health care policies such as targeted screening and preventive actions to be developed and implemented, especially now, in the post-COVID-19 era.

Malta has one of the highest prevalence rates of obesity in Europe. Indeed, ‘Xjenza Malta’, a governmental institution responsible for coordinating and promoting scientific research, in collaboration with the Ministry for Health and Active Aging, a branch of the Maltese government, has recently invested in an obesity research programme to enhance obesity research for better preventive evidence-based data [9]. A number of public health strategies such as “A Healthy Weight for Life” have been implemented by Public Health entities such as the Superintendence of Public Health Ministry for Health, the Elderly and Community Care to tackle this metabolic disease in the Maltese setting [10]. It is for this reason that obesity has been chosen as the focus of this study which aims to explore this condition within a subset of the Maltese population.

Establishing population characteristics and risk factors for obesity and its contributing factors requires a longitudinal cohort study in order to determine the temporal relationships between different risk factors and lifestyle choices [11]. A longitudinal cohort study following a health examination modality, has never been conducted in Malta and constitutes important and prestigious research by the University of Malta. It also represents a useful source of evidence-based prospective data that can be used by the state Public Health department and other stakeholders within the Maltese government as they engage in post-pandemic recovery plans. The study design can also be used as a framework for similar studies to be conducted in other small countries, islands, and regions.

## Methods

### SAHHTEK study population

The original SAHHTEK study (Ethics Ref. No: 19/2014) was a national cross-sectional study. The sample population for SAHHTEK was obtained by following a single stage randomisation and stratification process according to age (18–70 years), gender and locality (Malta and Gozo) using data from the identity cards and passport registry. Inclusion criteria included participants living in Malta for at least 6 months. A total of 1861 participants took part in the SAHHTEK study, and data was weighted to reflect the target population and adjust for non-response. The protocol for the original SAHHTEK study was published in 2016 [3].

### SAHHTEK 2.0 nested cohort sample population

The follow-up study currently being conducted, ‘SAHHTEK 2.0,’ follows a subset of SAHHTEK's population in the form of a longitudinal cohort study. Cross-linkage with the Malta National Mortality Register was performed with the help of the Directorate of Health Information and Research (DHIR) in Malta to identify any deceased participants of the SAHHTEK study and exclude them from the randomized selection process [12].

SAHHTEK 2.0 was designed to explore associations between a wide range of socio-demographic, socio-economic, lifestyle, clinical, and biological factors and adiposity outcomes over time. Sample-size considerations were based on the anticipated precision of interval estimates. Assuming a population proportion of 50% and a finite source population of 1861, a sample of 319 completed assessments was estimated to provide a 95% confidence interval with an absolute margin of error of approximately ±5 percentage points. Independently, for follow-up recruitment, the original cohort was ordered by age, gender and locality, and one in every four participants was systematically selected for invitation, yielding 455 individuals.

The precision of estimates will depend on the achieved follow-up sample size. For illustration, if approximately 50% of the 455 invited individuals participate, yielding approximately 228 completed assessments, a population proportion of 50% would be estimated with an approximate 95% confidence interval margin of error of ±6 percentage points.

### Participant recruitment

All SAHHTEK (first wave) participants had consented to be recontacted for future research studies by the principal investigator at the time. Participants are re-invited to participate in this follow-up study through an invitation letter sent by post where they are asked to contact the primary investigator by phone if they are interested. Participants who accept the invitation and who do contact the primary investigator are given an appointment and invited to the nearest of three government health clinics. Reminders in the form of text messages are sent to each participant a day prior to their appointment to increase attendance.

### Consent

Informed written consent is obtained for each participant on arrival at the governmental health clinic. The consent form is available in both Maltese and English and participants are given time to read the entire consent form and ask any questions they may have. The consent form outlines the key aspects of the study, including that participation is voluntary and that the research constitutes a follow-up assessment of metabolic health among adults in Malta. It details the duration and procedures of the study visit and specifies that all data are collected and stored in a pseudo-anonymised manner in accordance with General Data Protection Regulation (GDPR) requirements. Participants are informed of the minimal risks associated with venepuncture and the direct benefit of receiving their individual health results. Consent also includes agreement to be contacted for potential participation in future related research studies, and that participants retain the right to withdraw at any time without consequence.

### Ethics and logistics of follow-up

#### Ethical considerations and approval

The University Research Ethics Committee (UREC) granted ethical approval for this follow-up study (MED-2023-00078). All participants of the original SAHHTEK study (first wave) had explicitly consented, in writing, to be contacted for future follow-up studies of this nature.

#### Funding

The Maltese Ministry for Education, Sport, Youth, Research, and Innovation (MEYR) funded this work. The University of Malta Research Innovation and Development Trust (RIDT) was responsible for approving the use of funds as well as handling transactions.

#### General logistics

Permission to conduct study sessions at public government health clinics was obtained from the relevant authorities following ethical approval. A designated clinical area equipped with an examination couch for venepuncture and facilities ensuring participant privacy during measurements was required. Many study sessions were conducted on weekends and public holidays, when a reduced number of clinics operate in these governmental settings, allowing rooms to be made available for research purposes. However, additional weekday sessions at alternate venues were conducted to accommodate participants who expressed interest but were unable to attend during weekends or public holidays. When participants indicated that they were unavailable for their initially scheduled appointment, the primary investigator proactively discussed alternative logistical arrangements to facilitate participation, and reasonable requests were accommodated whenever possible. Data collection sessions were therefore coordinated according to the availability of participants, the principal investigator, and clinic facilities. Trained medical students were recruited to assist with data collection.

### Data collection

Data collection is ongoing at the time of writing and started in May 2024. The data collection period has been extended to longer than initially predicted as sessions could only be held on a limited number of days per month in view of participant, researcher, and clinic space availability. A questionnaire which collects data pertaining to socio-demographics, socio-economic, lifestyle, diet, alcohol consumption, smoking, sleeping patterns, medical history, COVID infection, COVID vaccination and long COVID symptoms was administered once the consent form is signed. In view of the increasing availability and use of pharmacological therapies for weight management, the medical history section of the questionnaire includes specific items on current use of weight-loss medications. Internationally-validated tools such as the Epworth Sleepiness Scale, Patient Health Questionnaire (PHQ-9) and Post-COVID-19 Functional Status (PCFS) scale, were incorporated into the questionnaire. These tools are used internationally and demonstrate good reliability and validity across various populations and research settings. The rest of the questionnaire is almost identical to the original SAHHTEK questionnaire to allow for comparison of the two research cohorts over time [3]. A copy of the questionnaire used in English has been uploaded with the supplementary material. A secure password-protected Google form was used to input questionnaire responses using pre-defined fields where possible to avoid human inputting errors, with access to answers restricted solely to the principal investigator.

A physical examination was conducted after the questionnaire. This included measuring participants' blood pressure, waist circumference, height, and weight, maintaining participant privacy throughout. Blood pressure was taken three times using a manual validated sphygmomanometer with participants' arms at the level of their hearts. Waist circumference was measured by positioning the measuring tape midway between the lower rib margin and the iliac crest. The participant was asked to breathe normally, and the reading was taken at the end of a light exhalation and was recorded to the nearest millimetre.

Height was measured by first asking participants to remove their shoes, heavy outer garments, and any hair ornaments or headdresses. They were asked to stand so that the back of the head, shoulder blades, buttocks, and heels were touching or aligned with the stadiometer which was situated against a wall. Participants were positioned in a natural, upright stance, with weight evenly distributed on both feet and arms hanging loosely at their sides. Participants were then instructed to keep their eyes focused straight ahead, to take a deep breath, and to stretch to their full height. The stadiometer headpiece was lowered onto the head so that the hair was pressed flat, and height was recorded to the nearest millimetre. Participants' weight was measured next. They were instructed to stand in the centre of the weighing scale platform with arms hanging loosely at their sides and to look straight ahead. The weight was then recorded in kilograms.

Finally, blood samples for complete blood count, renal profile, fasting blood glucose, lipid profile, HbA1C and liver function tests were collected. Blood samples for each participant were labelled and maintained at controlled room temperature following collection. In warmer months, insulated carriers with cooling packs were used solely to prevent temperature elevation above standard room temperature, without direct contact between samples and ice. This approach ensured comparable pre-analytical temperature conditions across seasons. All samples were transported to the laboratory within 2 h of collection to preserve sample integrity and minimise haemolysis [13].

Incidental findings such as high blood pressure or deranged blood results were explained to participants by the primary investigator and they were guided accordingly. Referral pathways include referral to the local emergency department in emergent cases or advising that patients seek advice from a specialist or family doctor as required. At the time of writing, a total of 268 participants, out of the 455 sample population had accepted to participate in the study.

## Equipment

### Blood pressure

Blood pressure was measured using the Welch Allyn® aneroid sphygmomanometer model 7670–04, the same device used in the SAHHTEK study. This model meets grade B performance standards based on clinical validation by the British Hypertension Society [14].

### Height and weight

Body weight (in kilograms) and height (in centimetres) were recorded using the ADAM® MDW-250 L digital health scale with height rod, also previously used in the SAHHTEK study [3].

## Data analyses

### General analytical approach

All statistical analyses will be conducted using IBM SPSS Statistics version 32. The analyses described constitute the prespecified analyses for the longitudinal component of SAHHTEK 2.0. Analyses not specified in this protocol that are subsequently undertaken in response to features of the observed data will be identified explicitly as exploratory or post hoc in subsequent research.

Participant flow will be reported, including the number of participants in the original cohort, the number selected for invitation to follow-up, the number contacted, the number participating, and the number included in each analysis.

### Adiposity outcomes

The primary outcome will be body mass index (BMI) measured in 2026 and expressed continuously in kg/m^2^. Secondary continuous adiposity outcomes will be waist circumference, measured in centimetres, and body weight, measured in kilograms, at the 2026 follow-up.

For each continuous follow-up outcome, its corresponding 2016 measurement will be included as a covariate. Thus, models of BMI in 2026 will include BMI in 2016; models of waist circumference in 2026 will include waist circumference in 2016; and models of weight in 2026 will include weight in 2016. The resulting estimates will therefore represent associations with the 2026 outcome conditional on the corresponding baseline adiposity measure.

Absolute change in BMI will be calculated as: Delta BMI = (BMI 2026) – (BMI 2016) and examined as a prespecified secondary outcome. Obesity will be defined as BMI ≥30 kg/m^2^.

### Exposure definitions

The primary longitudinal analyses will use exposures measured in 2016.

Age will be entered continuously in completed years. Gender will be entered as a categorical variable with two levels, male and female.

Educational attainment will be entered categorically using seven prespecified levels: no formal education, primary education, unfinished secondary education, completed secondary education, sixth form, undergraduate education, and second-stage tertiary education.

Locality will be represented categorically using six geographical areas: Northern Harbour, Southern Harbour, South Eastern, Western, Northern, and Gozo.

Smoking status will be represented categorically as never smoker, former smoker, current smoker, and passive smoker, according to the definitions contained in the study questionnaire. Never smoker will be the reference category.

Alcohol consumption will be represented using two separate variables. Drinking frequency will be categorized as rare/non-drinker, occasional drinker, and frequent drinker, with rare/non-drinker as the reference category. Average alcohol consumption will be categorized as <1, 1–2, and > 2 alcohol units/day, with <1 unit/day as the reference category. Drinking frequency and average units/day will be evaluated in separate models.

Physical activity will be represented by total moderate-to-vigorous physical activity (MVPA), expressed in minutes/week and derived from reported moderate and vigorous activity. Walking will be represented separately as minutes/week.

Mediterranean dietary adherence will be represented by the predefined Mediterranean diet score ranging from 0 to 18. The score comprises nine components—fruit, vegetables, legumes, cereals, fish, meat and meat products, dairy products, alcohol, and olive oil—each scored from 0 to 2 according to the predefined scoring thresholds. Higher scores indicate greater adherence to the specified Mediterranean dietary pattern. The score will be entered continuously in the primary analysis.

Sleep duration will be represented categorically as <6 h, 6–9 h, and > 9 h, with 6–9 h as the reference category. Daytime sleepiness will be represented continuously using the total Epworth Sleepiness Scale score.

Clinical and biochemical exposures will comprise systolic blood pressure, diastolic blood pressure, fasting glucose, total cholesterol, LDL cholesterol, HDL cholesterol, and triglycerides measured in 2016. Blood pressure will be expressed in mmHg and calculated as the arithmetic mean of the three readings obtained at the assessment. Fasting glucose, total cholesterol, LDL cholesterol, HDL cholesterol, and triglycerides will be expressed in mmol/L. These variables will be treated as continuous.

### Primary longitudinal analysis

The primary longitudinal analyses comprise a set of prespecified exposure–outcome models. No formal adjustment for multiple comparisons will be applied. Interpretation will therefore emphasize the magnitude, direction and precision of estimated associations, together with their consistency across related analyses, rather than statistical significance in isolation. Findings will be interpreted in the context of the number of associations examined. Separate multivariable linear regression models will be fitted for each prespecified baseline exposure, with BMI in 2026 as the primary dependent variable.

The general form of the primary model will be:

$$\mathrm{BM}I_{2026}=\beta_{0}+\sum_{n}\beta_{n}X{\left(2016\right)}_{n}+\beta_{2}\mathrm{BM}I_{2016}+\sum_{k}\beta_{k}C_{k}+\varepsilon$$

where:

$\mathrm{BM}I_{2026}$= BMI in 2026.

$\sum_{n}\beta_{n}X{\left(2016\right)}_{n}$= the baseline exposure term under investigation, where \(X(2016)\) represents the prespecified 2016 exposure variable or set of indicator terms required to represent that exposure. A separate regression model will be fitted for each prespecified exposure listed in Table 1.

**Table 1:** Prespecified primary longitudinal models for BMI in 2026.

Model	Baseline exposure (2016)	Prespecified coding
A01	Smoking status	Categorical: never, former, current, passive; never smoker as reference
A02	Alcohol drinking frequency	Categorical: rare/non-drinker, occasional, frequent; rare/non-drinker as reference
A03	Alcohol consumption	Categorical: 〈1,1−2,〉2 units/day; <1 unit/day as reference
A04	Moderate-to-vigorous physical activity (MVPA)	Continuous, minutes/week
A05	Walking	Continuous, minutes/week
A06	Mediterranean diet score	Continuous, range 0–18; higher score indicates greater adherence
A07	Sleep duration	Categorical: <6, 6–9, >9 h; 6–9 h as reference
A08	Epworth Sleepiness Scale	Continuous, total score, range 0–24
A09	Systolic blood pressure	Continuous, mmHg; arithmetic mean of three readings
A10	Diastolic blood pressure	Continuous, mmHg; arithmetic mean of three readings
A11	Fasting glucose	Continuous, mmol/L
A12	Total cholesterol	Continuous, mmol/L
A13	LDL cholesterol	Continuous, mmol/L
A14	HDL cholesterol	Continuous, mmol/L
A15	Triglycerides	Continuous, mmol/L
A16	Age	Continuous, completed years
A17	Gender	Categorical: male/female
A18	Educational attainment	Categorical: no formal education, primary education, unfinished secondary education, completed secondary education, sixth form, undergraduate education, second-stage tertiary education
A19	Locality	Categorical: Northern Harbour, Southern Harbour, South Eastern, Western, Northern, Gozo

${\mathrm{BMI}}_{2016}$= baseline BMI.

$C_{k}$= prespecified adjustment covariates, as defined in the analysis plan.

$\beta_{0}$= intercept.

$\beta_{k},\beta_{n}$= regression coefficients.

ε= error term.

The initial core adjustment set will comprise baseline BMI, age, gender, educational attainment, and locality. Age and baseline BMI will be entered continuously; gender, education, and locality will be entered categorically. Where one of these variables is itself the exposure under investigation, it will not additionally be included as a duplicate adjustment variable. The core adjustment set was specified a priori based on subject-matter knowledge and informed by a directed acyclic graph (DAG), Fig. 1, representing the hypothesised causal relationships between the baseline exposures, potential confounders, and subsequent adiposity.

**Fig. 1:**
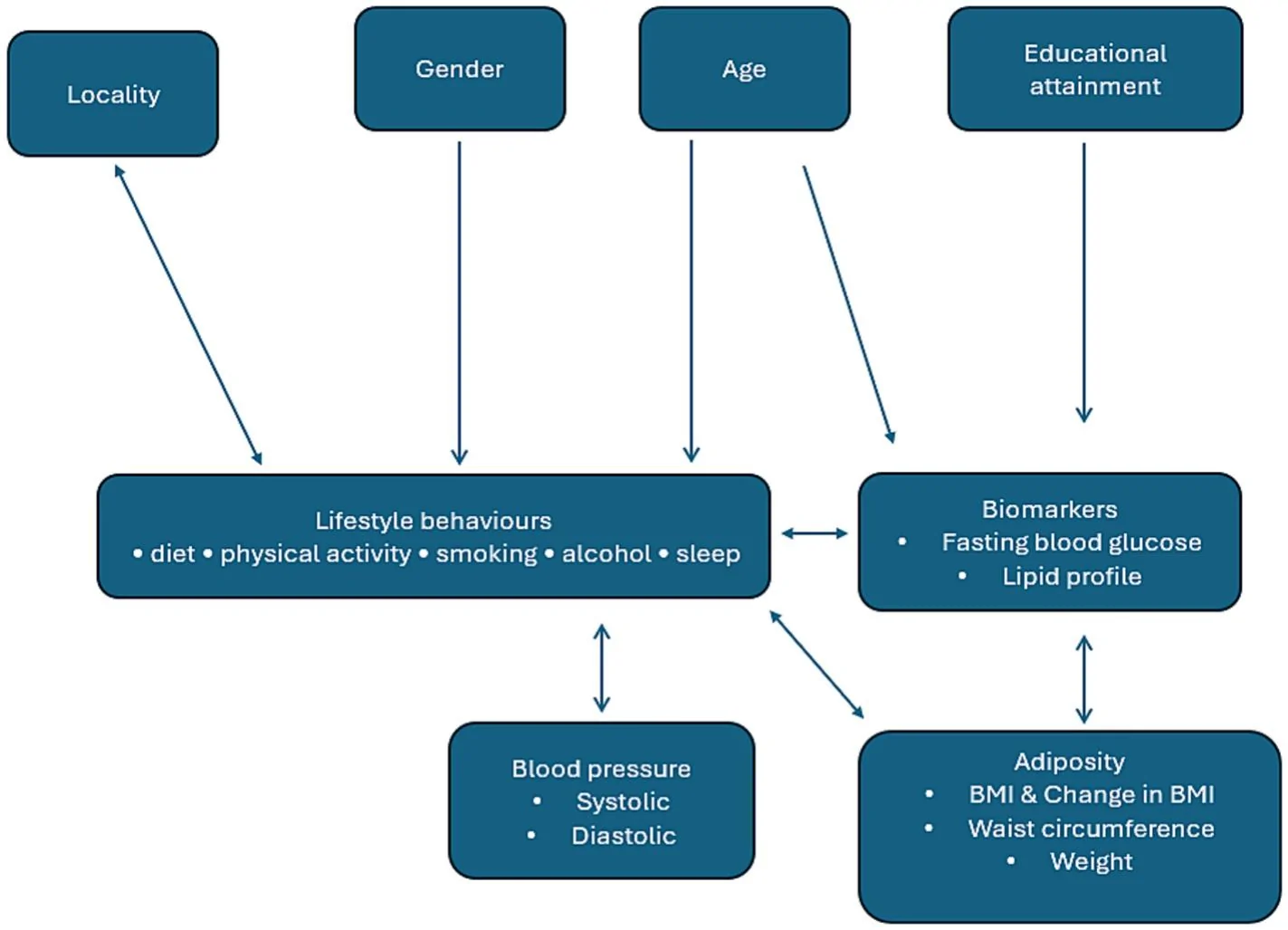

The prespecified primary longitudinal models are shown in Table 1.

### Prespecified primary longitudinal models for BMI in 2026

Primary longitudinal models will be adjusted for baseline BMI (2016), age, gender, educational attainment, and locality. Baseline BMI and age will be entered as continuous variables, while gender, educational attainment, and locality will be entered as categorical variables. Where age, gender, educational attainment, or locality is itself the exposure under investigation, that variable will not additionally be included as an adjustment variable. These prespecified adjustment variables will be retained in the respective models irrespective of their observed associations with the outcome.

Assumptions of the linear regression models will be assessed using appropriate graphical and numerical diagnostics. These will include examination of the functional form of continuous predictors, residual distributions, homoscedasticity and potentially influential observations. Where marked departures from model assumptions are identified, appropriate sensitivity analyses or alternative specifications may be considered. Any modification not prespecified in this protocol will be identified as exploratory or post hoc. Continuous exposures will remain modelled linearly in the primary analysis; alternative functional forms will be examined only as sensitivity analyses where there is clear evidence of important non-linearity.

The core adjustment set will comprise baseline BMI, age, gender, educational attainment, and locality, as specified above. Where age, gender, educational attainment, or locality is itself the exposure under investigation, that variable will enter the model as the exposure and will not also be entered separately as an adjustment covariate. The remaining variables in the core adjustment set will be retained. For example, a model examining gender as the exposure will adjust for baseline BMI, age, educational attainment, and locality.

Exposures recorded as continuous variables will be used as such, without categorisation. Regression coefficients and 95% confidence intervals will represent the adjusted difference in 2026 BMI associated with a prespecified and clinically or epidemiologically interpretable increase in the exposure. The reporting increments will be defined in results tables. Suitable reporting increments may include 5 years for age, 60 min/week for MVPA and walking, 1 point for the Mediterranean diet score and Epworth Sleepiness Scale, 10 mmHg for systolic and diastolic blood pressure, and 1 mmol/L for biochemical measurements.

### Secondary longitudinal analyses

The prespecified exposure models in Table 1 will additionally be applied to waist circumference and body weight as secondary continuous outcomes. For waist circumference, 2026 waist circumference will be the dependent variable and 2016 waist circumference will replace baseline BMI as the baseline outcome covariate. For body weight, 2026 weight will be the dependent variable and 2016 weight will be included as the corresponding baseline outcome covariate.

Absolute change in BMI between 2016 and 2026 will also be examined as a secondary continuous outcome, calculated as ΔBMI = BMI in 2026 − BMI in 2016. Separate multivariable linear regression models will be fitted for each prespecified baseline exposure, with ΔBMI as the dependent variable. These models will adjust for baseline BMI, age, gender, educational attainment, and locality, subject to the same exposure-specific adjustment rules described for the primary analyses. The change-score analysis will be interpreted as a secondary analysis complementary to the primary ANCOVA-style analysis of 2026 BMI adjusted for 2016 BMI.

### Prespecified effect-modification analyses

Prespecified effect modification by gender will be assessed for the following baseline lifestyle exposures: smoking status, average alcohol consumption, MVPA, Mediterranean diet score, and sleep duration.

For each analysis, the corresponding primary longitudinal model will be extended by including an exposure-by-gender interaction term. The exposure main effect and gender main effect will remain in the model in accordance with the hierarchical principle, together with the remaining covariates specified for the corresponding primary exposure model.

Interaction terms will be evaluated and retained in the model and their 95% confidence intervals will be reported. Where appropriate, exposure associations will additionally be presented stratified by gender to facilitate interpretation. Effect-modification analyses will be considered secondary analyses.

### Assessment of follow-up participation

Given that complete baseline data from 2016 are available for the 455 individuals selected for invitation to the 2026 follow-up, potential selection associated with follow-up participation will be assessed directly.

Baseline characteristics as at 2016 of individuals who participate and those who do not participate in the 2026 follow-up will be described. Prespecified variables will include age, gender, education, locality, BMI, weight, waist circumference, smoking, alcohol consumption, MVPA, walking, Mediterranean diet score, sleep duration, Epworth Sleepiness Scale score, systolic and diastolic blood pressure, fasting glucose, total cholesterol, LDL cholesterol, HDL cholesterol, and triglycerides.

Differences between participants and non-participants will be quantified using standardized differences where appropriate. These comparisons will focus on the magnitude and direction of differences. The observed pattern of differences will be used to characterize the specific potential for selection associated with follow-up participation.

Because the 2026 outcome is unavailable for individuals who do not participate in follow-up, comparison of baseline characteristics cannot by itself eliminate potential selection bias arising from loss to follow-up. The participant–non-participant comparisons will therefore be used to characterize the direction and magnitude of observed selection rather than to establish the absence of selection bias.

#### Missing data

Missingness will be summarized for each analysis variable. Participants without a valid measurement of the relevant 2026 outcome will not contribute to that outcome analysis. The primary regression analyses will use complete cases with respect to the exposure, outcome, baseline outcome measure, and adjustment covariates required for the corresponding model. The number of participants included in each model will be reported.

#### Reporting of estimates

Results will emphasize estimated associations and their 95% confidence intervals. Confidence intervals will be used to convey the magnitude and precision of estimates with respect to random variability.

For linear regression models, regression coefficients and 95% confidence intervals will be reported. For continuously measured exposures, coefficients will be interpreted according to exposure increments; for categorical exposures, estimates will be interpreted relative to the prespecified reference category.

Interpretation of an estimate will take account of the temporal ordering of exposure and outcome measurements, the adjustment strategy, study design, and assumptions relevant to the particular research question.

#### Reproducibility and deviations from the analysis plan

The prespecified statistical analysis will be carried out using SPSS version 32. The models will be run using the provided software menus.

Where a methodological or data-related issue subsequently necessitates a change in the analysis protocol, the resulting publication will identify the originally prespecified analysis, the modification, the reason for the change, and whether the decision was made before or after examination of the relevant results. Additional analyses may be undertaken where scientifically informative, however analyses not specified prospectively will be explicitly identified as exploratory or post hoc.

### Study limitations

The principal potential source of selection in SAHHTEK 2.0 is non-participation at the 2026 follow-up. Of the original 2016 cohort, 455 individuals were systematically selected for invitation to follow-up, and not all invited participants are expected to participate. Selection would be of particular concern if participation is associated with baseline adiposity or with the socio-demographic, lifestyle, or metabolic characteristics examined as exposures. This will be assessed directly because 2016 measurements are available for all 455 individuals selected for follow-up, including those who do not participate in 2026. Baseline characteristics of participants and non-participants will therefore be compared using standardized differences. The magnitude and direction of these differences will be considered when interpreting the potential for selection associated with follow-up participation.

A second concern is error in the measurement of lifestyle exposures. Smoking, alcohol consumption, physical activity, diet, and sleep are questionnaire-derived measures and therefore depend on participants' reporting. Misclassification of these exposures could attenuate, exaggerate, or otherwise alter estimated associations, depending on the nature of the measurement error. To mitigate this, exposure definitions and scoring procedures have been prespecified. These include predefined smoking and alcohol categories, moderate-to-vigorous physical activity and walking expressed in minutes/week, the predefined 0–18 Mediterranean diet score, prespecified sleep-duration categories, and the Epworth Sleepiness Scale score.

The availability of measurements at two time points, 2016 and 2026, permits assessment of long-term differences in adiposity but does not permit the trajectory of change between these occasions to be determined. The analyses therefore concern adiposity measured at follow-up, conditional on baseline adiposity, and change between the two observed measurement occasions; they will not be interpreted as describing the shape or timing of adiposity trajectories during the intervening decade.

### Public health relevance and policy implications

Obesity represents a major and growing public health challenge in Malta and worldwide, with substantial implications for non-communicable disease burden, healthcare utilisation, and health inequalities. By providing longitudinal data on changes in adiposity and their associations with socio-demographic, socioeconomic, lifestyle, and clinical factors over a 10-year period, this study will generate evidence that can inform population-level prevention strategies and targeted interventions.

Recent research further highlights the broader relevance of lifestyle factors captured in the present study. An umbrella review on physical activity and the prevention of mental health complications has demonstrated consistent protective effects of regular physical activity across multiple mental health outcomes, underscoring the importance of physical activity patterns when examining obesity and related determinants [15]. Given that the current study collects detailed lifestyle data, future analyses could explore similar multidimensional health implications within this cohort. In addition, national trend analyses conducted before and during the COVID-19 pandemic [16], illustrate methodological approaches for assessing how major societal disruptions may influence population health outcomes over time. These studies provide a useful framework for interpreting temporal trends and pandemic-related effects and suggest avenues for future research using longitudinal or repeated cross-sectional data to better understand post-pandemic health trajectories.

The findings are expected to support national policy development by identifying modifiable risk factors and vulnerable population subgroups, thereby guiding resource allocation, surveillance priorities, and the design of obesity prevention and health promotion programmes. Besides contributing to national health policy, this study is also relevant to the European Union cardiovascular health plan: The Safe Hearts Plan, as it ties in to shifting focus to prevention, early detection, and personalized care, while tackling risk factors like obesity, smoking, and poor diet through integrated strategies [17].

During the preparation of this work the authors used ChatGPT in order to enhance grammar and clarity of certain aspects of the manuscript. After using this tool, the authors reviewed and edited the content as needed and take full responsibility for the content of the published article.

## CRediT authorship contribution statement

**Elizabeth Grech:** Writing – review & editing, Writing – original draft, Resources, Project administration, Methodology, Investigation, Formal analysis, Data curation, Conceptualization. **Neville Calleja:** Supervision, Methodology, Formal analysis, Conceptualization. **Keith Calleja:** Writing – review & editing, Methodology, Data curation. **Axel Tonna:** Writing – review & editing, Methodology, Data curation. **Grazia Magro:** Writing – review & editing, Methodology, Data curation. **Sarah Cuschieri:** Writing – review & editing, Writing – original draft, Supervision, Methodology, Formal analysis, Data curation, Conceptualization.

## Funding

This work was supported by the Ministry for Education, Sports, Youths, Research and Innovation (MEYR), Malta.

## Declaration of competing interest

The authors declare the following financial interests/personal relationships which may be considered as potential competing interests: Elizabeth Grech reports financial support was provided by Ministry for Education, Sports, Youths, Research and Innovation (MEYR), Malta. If there are other authors, they declare that they have no known competing financial interests or personal relationships that could have appeared to influence the work reported in this paper.
