# Supplementary material for: Rationale and methodology of an ongoing Maltese longitudinal cohort study focusing on obesity: a decade of population health surveillance
Source: Glob Epidemiol. 2026 Nov 4;12:100298. doi: 10.1016/j.gloepi.2026.100298 (PMC13770603; doi:10.1016/j.gloepi.2026.100298)
Supplement: Supplementary file 1 [file mmc1.pdf]

Analysis of the metabolic status of the Maltese population: a nested cohort study

Adapted from EHIS, 2019/2020 and previous SAHHTEK questionnaire

INDEX NUMBER ______________________

Section A: Socio-demographic

1. SD 1. What is your gender?

   Mark only one oval.

   - Male
   - Female
   - Prefer not to say

2. SD 2. What is your age as of your last birthday?

3. SD 3. Which locality do you currently reside in?

   Mark only one

   - *Ħ'Attard (ATD)*
   - *Ħal Balzan (BZN)*
   - *Il-Bidnija (MST)*
   - *Il-Birgu (Vittoriosa) (BRG)*
   - *Birkirkara (BKR)*
   - *Birżebbuġa (BBĠ)*
   - *Bormla (Cospicua) (BML)*
   - *Ħad-Dingli (DGL)*
   - *Il-Fgura (FGR)*
   - *Il-Furjana (FRN)*
   - *Ħal Għargħur (GĦR)*
   - *Ħal Għaxaq (GXQ)*
   - *Il-Gudja (GDJ)*
   - *Il-Gżira (GŻR)*
   - *Il-Ħamrun (ĦMR)*
   - *L-Iklin (IKL)*
   - *Il-Kalkara (KKR)*
   - *Ħal Kirkop (KKP)*
   - *Ħal Lija (LJA)*
   - *Ħal Luqa (LQA)*
   - *Il-Marsa (MRS)*
   - *Marsaskala or Wied il-Għajn (MSK)*
   - *Marsaxlokk (MXK)*
   - *L-Imdina (MDN)*
   - *Il-Mellieħa (MLĦ)*
   - *L-Imġarr (MĠR)*
   - *Il-Mosta (MST)*
   - *L-Imqabba (MQB)*
   - *L-Imsida (MSD)*
   - *L-Imtarfa (MTF)*
   - *In-Naxxar (NXR)*

- *Raħal Ġdid (Paola) (PLA)*
- *Pembroke (PBK)*
- *Tal-Pietà (PTA)*
- *Ħal Qormi (QRM)*
- *Il-Qrendi (QRD)*
- *Ir-Rabat, Malta (RBT)*
- *Ħal Safi (SFI)*
- *San Ġwann (SĠN)*
- *Santa Luċija (SLĊ)*
- *Santa Venera (SVR)*
- *L-Isla (Senglea) (ISL)*
- *Is-Siġġiewi (SĠW)*
- *Tas-Sliema (SLM)*
- *San Ġiljan (STJ)*
- *San Pawl il-Baħar (SPB)*
- *Is-Swieqi (SWQ)*
- *Ħal Tarxien (TXN)*
- *Ta' Xbiex (XBX)*
- *Il-Belt Valletta (VLT)*
- *Ix-Xgħajra (XJR)*
- *Ħaż-Żabbar (ŻBR)*
- *Ħaż-Żebbuġ (Malta) (ŻBĠ)*
- *Iż-Żejtun (ŻTN)*
- *Iż-Żurrieq (ŻRQ)*
- *Il-Fontana or It-Triq tal-Għajn (FNT)*
- *Għajnsielem (GSM)*
- *L-Għarb (GRB)*
- *L-Għasri (GSR)*
- *Ta' Kerċem (KĊM)*
- *Il-Munxar (MXR) and Ix-Xlendi (XLN)*
- *In-Nadur (NDR)*
- *Il-Qala (QLA)*
- *Ir-Rabat(VCT)*
- *San Lawrenz (SLZ)*
- *Ta' Sannat (SNT)*
- *Ix-Xagħra (XRA)*
- *Ix-Xewkija (XWK)*
- *Iż-Żebbuġ (Gozo) (ŻBB) and Marsalforn (MFN)*

4. SD 4. What is the highest level of education that you have successfully completed?
Mark only one.

- No formal education/Pre-primary
- Primary
- School for persons with a disability
- Secondary (general)
- Foundation or Introductory courses at MCAST of one (1) year or less
- Secondary (vocational) E.g. Trade School
- Post-secondary (general) E.g. Junior College
- Post-secondary (vocational) completed before the year 2000 (excluding ITS)

- Post-secondary (vocational) courses of less than two (2) years E.g. MCAST/MCAST National Diploma, ITS or similar institutions
- Post-secondary (vocational) courses two (2) years or more E.g. MCAST/MCAST National Diploma, ITS or similar institutions
- University level diploma/certificate or MCAST Higher National Diploma
- Bachelor's degree or equivalent
- University of Malta, MCAST or other tertiary level institutions
- Postgraduate Diploma/Certificate
- Master's Degree
- Doctorate (PhD/DBA)
- Other: ______________________

5. SD 5. What is your current labour status?

Mark only one oval.

- Employed (including unpaid work for a family business or holding, an apprenticeshipor paid traineeship, currently not at work due to maternity, parental, sick leave or holidays)
- Unemployed
- Pupil or student
- Retired
- Unable to work due to long standing health problems
- Cannot work due to disability
- Take care of house or family
- Community service as ordered by court

Nature of Employment

6. SD. 6 In your main job do you work full-time or part time?

Mark only one oval.

- Full time
- Part time

Marital Status

7. SD. 7 What is your CURRENT legal marital status?

Mark only one oval.

- Single (never married or never in a registered partnership)
- Married (or in a registered partnership)
- Legally separated
- Widowed (or persons whose registered partnership ended with the death of the partner)
- Divorced (or persons whose registered partnership was legally dissolved)

- [ ] Annulled

Section B: Lifestyle, Smoking

8. LS. 1 Do you smoke any tobacco products (excluding electronic cigarettes or similar electronic devices)?

'Current smoker' is defined as someone who smoked more than one cigarette per day at the time of the interview or who has ***quit smoking within in the last year.***

'Former smoker' is defined as someone who used to smoke cigarettes regularly or had smoked at least 100 cigarettes in the past but who ***quit smoking at least one year ago****.*

'Passive smoker' is defined as someone who is currently being exposed to second hand smoke at home or at work for at least ***30 minutes per day for a minimum of 8 weeks****.*

'*Never smoker' is a person who has never smoked at all or who has smoked less than 100 cigarettes in their lifetime.*

Mark only one oval.

- [ ] Current smoker
- [ ] Ex-smoker
- [ ] Passive smoker
- [ ] Never smoker

9. LS 2. Do you currently use electronic cigarettes or similar electronic devices?

E.g. Electronic-shisha, electronic pipe

Mark only one oval.

- [ ] Yes, daily vaping
- [ ] Yes, occasionally vaping
- [ ] No, but I did in the past
- [ ] Never vaped

LS 3. - Alcohol Consumption

10. How often do you drink alcohol?

Mark only one oval.

- [ ] At least three days a week (frequent drinker)
- [ ] At least once a week or once a month (occasional drinker)
- [ ] I do not drink alcohol/only once or twice in the past 12 months (rare/non-drinker)

11. N 8. How many units of ALCOHOL do you consume in a **day**?

This question refers to alcohol units per day, if someone drinks on weekends and has, for example 4 units at the weekend, that would mean they drink less than 1 unit per day (average them out).

1 Alcohol Unit (AU) = 12 g

Mark only one oval.

- <1 U.A./day
- 1–2 U.A./day
- >2 U.A./day

LP – Physical activity

· *Next, I am going to ask you about the time you spend doing different types of physical activity in a typical week.*

· *In this section, when referring to work, keep in mind any sort of paid/unpaid work (e.g. housework, studying or training etc.) is included.*

12. LP. 1 Before COVID-19, when you were working, which one of the following best described you?

*In this section, when referring to work, keep in mind any sort of paid/unpaid work (e.g. **housework**, studying or training etc.) is included.*

Mark only one oval.

- Mostly sitting or standing
- Mostly walking or tasks of moderate physical effort
- Mostly heavy labour or physically demanding work
- Not performing any working tasks

13. LP. 2 Since COVID-19, when you are working, which one of the following best describes what you do?

*In this section, when referring to work, keep in mind any sort of paid/unpaid work (e.g. **housework**, studying or training etc.) is included.*

Mark only one oval.

- Mostly sitting or standing
- Mostly walking or tasks of moderate physical effort
- Mostly heavy labour or physically demanding work
- Not performing any working tasks

14. LP. 3 During the last 7 days, on how many days did you carry out vigorous physical activities?

Please use the 'other' option to input number of days in the past week the participant has carried out vigorous physical activities.

Mark only one oval.

- Don't know
- None
- Other: ____________________

Examples of Vigorous Activities

Vigorous activities		
Jogging, Running	Basketball	Aerobics
Carrying loads, >22Kg	Judo, Karate, Kick boxing	Jumping robe
Bicycling fast (>19.3Km/hr)	Stair climbing/ Stairmaster	Football
Organized sports classes	Swimming laps	Tennis

15. LP. 4 During the past 7 days, how much time did you spend in total doing **vigorous** physical activities?

*Please use the 'other' option to indicate how much time **IN MINUTES** the participant has spent in total doing vigorous physical activities in the past 7 days.*

Mark only one oval.

- Don't know
- None
- Other: ____________________

16. LP 5 During the past 7 days, how much time did you spend in total doing **moderate** physical activities?

*Please use the 'other' option to indicate how much time **IN MINUTES** the participant has spent in total doing moderate physical activities in the past 7 days.*

Mark only one oval.

- Don't know
- None
- Other: ____________________

Examples of moderate physical activity

Moderate activities		
Gardening: Planting, weeding	Walking fast (5km/hr)	Lifting, turning, carrying <22kg
Housework: Mopping, sweeping, vacuuming	Bicycling (<19.3km/hr), Volleyball, Golf, Bowling	Carpentry
Playing with children: walking, kneeling, lifting	Aerobics low impact, Vigorous stretching, Yoga	Rowing, Sailing
Washing, working on car	Dancing	Weightlifting
Skateboarding		

17. LP. 6 During the past 7 days, how much time did you spend in total doing **mild** physical activities?

*Please use the 'other' option to indicate how much time **IN MINUTES** the participant has spent in total doing mild physical activities in the past 7 days.*

Mark only one oval.

- Don't know
- None
- Other: ____________________

18. LP. 7 During the past 7 days, on how many days did you walk for at least 10 minutes at a time?

Please use the 'other' option to indicate how many days the participant has spent in total walking for at least 10 minutes at a time.

Mark only one oval.

- Don't know
- None
- Other: ____________________

19. LP. 8 During the past 7 days, how much time did you spend walking in total?

*Please use the 'other' option to indicate how much time **IN MINUTES** the participant has spent walking*

Mark only one oval.

- Don't know
- None
- Other: ______________________

Section C: Nutrition

20. N. 1 How many portions of FRUIT do you eat in a **day**?

1 portion = 150g

Mark only one oval.

- <1 portion/day
- 1 - 1.5 portions/day
- >2 portions/day

21. N. 2 How many portions of VEGETABLES do you eat in a **day**?

1 portion = 100g

Mark only one oval.

- <1 portion/day
- 1 - 2.5 portions/day
- >2.5 portions/day

22. N. 3 How many portions of LEGUMES do you eat in a **week**?

1 portion = 70g
Examples of legumes: Lentils · Peas · Broad beans · Chickpeas · Soybeans · Beans

Mark only one oval.

- <1 portion/week
- 1 - 2 portions/week
- >2 portions/week

23. N. 4 How many portions of CEREALS do you eat in a **day**?

1 portion: 130 g
Common cereal foods include bread, breakfast cereals, rice, and pasta.

Mark only one oval.

- <1 portion/day
- 1 - 1.5 portions/day
- >1·5 portions/day

24. N. 5 How many portions of FISH do you eat in a **week**?

1 portion: 100 g

Mark only one oval.

- <1 portion/week
- 1–2·5 portions/week
- >2·5 portions/week

25. N. 6 How many portions of MEAT AND MEAT PRODUCTS do you eat in a **day**?

1 portion: 80 g

Mark only one oval.

- <1 portion/day
- 1–1·5 portions/day
- >1·5 portions/day

26. N 7. How many portions of DAIRY PRODUCTS do you eat in a **day**?

1 portion: 180 g

Mark only one oval.

- <1 portion/day
- 1–1·5 portions/day
- >1·5 portions/day

27. N 9. How often do you use OLIVE OIL in your food?

Mark only one oval.

- Occasional use
- Frequent use
- Regular use

28. N 10. How often is salt, stock/seasoning cubes or a salty sauce added in cooking or preparing foods in your household?

Mark only one oval.

- Never
- Rarely (once per week)
- Sometimes (2-4 times a week)
- Often (4-6 times per week)
- Always (daily)

29. N 11. Do you examine the label of processed packaged foods for salt content (either front of pack label or nutrient content panel?

Mark only one oval.

- Never
- Rarely (once per week)
- Sometimes (2-4 times a week)
- Often (4-6 times per week)
- Always (daily)

Questions on your sleep patterns

30. How many hours do you normally sleep at night when you must work the next day?

Mark only one oval.

- < 6 hours
- 6 - 9 hours
- > 9 hours
- I don't work

31. How many hours do you normally sleep at night when you don’t have to work the next day?

Mark only one oval.

- < 6 hours
- 6 - 9 hours
- > 9 hours

32. Do you have difficulties in sleeping through the night?
Mark only one oval.

- Yes
- No

Difficulty sleeping

33. How long have you been experiencing difficulties in sleeping through the night?
Mark only one oval.

- Since before the onset of COVID-19 pandemic
- Since COVID-19 pandemic
- Since acquiring the COVID infection
- Other: ____________________

Sleep

34. Have you even been told by a doctor or another health professional that you have a sleep disorder?
Mark only one oval.

- ( ) Yes
- ( ) No

35. How likely are you to doze off or fall asleep in the following situations, in contrast to feeling just tired? This refers to your usual way of life in recent times. Even if you have not done some of these things recently try to work out how they would have affected you.

Tick all that apply.

	Watching TV	Sitting, inactive in a public place (e.g. a theater or a meeting)	As a passenger in a car for an hour without a break	Lying down to rest in the afternoon when circumstances permit	Sitting and talking to someone	Sitting quietly after a lunch without alcohol	In a ca while stoppe for a few minute in the traffic
**would never doze**	☐	☐	☐	☐	☐	☐	☐
**slight chance of dozing**	☐	☐	☐	☐	☐	☐	☐
**moderate chance of dozing**	☐	☐	☐	☐	☐	☐	☐
**high chance of dozing**	☐	☐	☐	☐	☐	☐	☐

Section D1: Medical History

36. MH. 1 Have you ever been diagnosed with the following diseases or conditions by a medical doctor?

The option, 'None' can only be used on its own. NB: re - cancer: this refers to whether they have ever had cancer and does not take into account current remission etc.

Tick all that apply.

- [ ] None
- [ ] Asthma (including allergic asthma)
- [ ] Chronic bronchitis, chronic obstructive pulmonary disease (COPD), emphysema
- [ ] Myocardial infarction (heart attack) or chronic consequences of myocardial infarction
- [ ] Coronary heart disease (angina pectoris)
- [ ] High blood pressure (hypertension)
- [ ] Elevated blood cholesterol
- [ ] Stroke (cerebral haemorrhage, cerebral thrombosis) or chronic consequences of stroke
- [ ] Osteoarthritis (joint degeneration)
- [ ] Lower back disorder or other chronic back defect
- [ ] Neck disorder or other chronic neck defect
- [ ] Type 1 Diabetes Mellitus
- [ ] Type 2 Diabetes Mellitus
- [ ] Cirrhosis of the liver
- [ ] Kidney problems
- [ ] Osteoporosis
- [ ] Hypothyroidism
- [ ] Hyperthyroidism
- [ ] Cancer
- [ ] Chronic anxiety
- [ ] Chronic depression
- [ ] Anorexia/Bulimia Nervosa
- [ ] Other mental health conditions
- [ ] Other: ____________________

37. MH. 2 Are you currently taking any regular medication?

Mark only one oval.

- ( ) Yes
- ( ) No

Section D2: Types of Medication

38. MH. 3 Do you take any of the following medications?

#

Mark only one oval per row.

	Yes	No	Not sure
Anti-Hypertensive Medication (tal-pressjoni)	⬭	⬭	⬭
Cholesterol lowering agents (tal-cholesterol)	⬭	⬭	⬭
Oral antihyperglycaemic agents (taz-zokkor)	⬭	⬭	⬭
Medications for hypothyroidism (tat-thyroid baxx)	⬭	⬭	⬭
Medications for hyperthyroidism (tat-thyroid għoli)	⬭	⬭	⬭
Anti-depressive/Anxiety Medications	⬭	⬭	⬭
Anticoagulants/Antiplatelet agents (biex traqqaq id-demm)	⬭	⬭	⬭

39. MH. 4 List of medication classes being taken by participant if known.

Only write names of medications (trade or generic), no need to write doses or regimens.

____

____

____

____

____

Section D3: Medical History Cont.

40. MH. 5.1 In the past two weeks how often have you been bothered by any of the following concerns?

Tick all that apply.

	Not at all	Several Days	More than half the days	Nearly every day
**Bothered by having little interest or pleasure in doing things**	◯	◯	◯	◯
**Bothered by feeling down, depressed or hopeless**	◯	◯	◯	◯
**Bothered by trouble falling or staying asleep or sleeping too much**	◯	◯	◯	◯
**Bothered by feeling tired or having little energy**	◯	◯	◯	◯
**Bothered by having poor appetite or overeating**	◯	◯	◯	◯
**Bothered by feeling bad about yourself or that you are a failure or have let your family down**	◯	◯	◯	◯
**Bothered by trouble concentrating on things such as reading or watching the TV**	◯	◯	◯	◯
**Bothered by moving or speaking so slow that other people have noticed? Or being so fidgety or restless that you have been moving around a lot more than usual**	◯	◯	◯	◯
**Bothered by thoughts that you would be better off dead or of hurting yourself in some way**	◯	◯	◯	◯

Section E - COVID-19

41. C. 1 Have you taken the COVID-19 vaccine?

Mark only one oval.

- No
- Yes - took 1 dose (not J&J) and did not take booters
- Yes - full dose (2 vaccines in total) or 1 dose if J&J
- First booster (3 vaccines in total) or 2 doses if J&J
- Second booster (4 vaccines in total) or 3 doses if J&J
- More than 4 vaccine doses or more than 3 doses if J&J
- Yes, but I have lost track of how many vaccines I have taken

42. C. 2 Have you ever had COVID-19 infection?

Mark only one oval.

- Yes (Confirmed by positive PCR or RAT)
- No (Never had positive COVID test)

43. Date of first positive COVID test

____

44. Section F1 - Long COVID

LC. 1 How many times have you been infected with COVID-19? (confirmed by a positive PCR or RAT)

Mark only one oval.

- Once
- Twice
- Three times
- Four times
- Five times
- More than five times

Section F2 - Post-COVID-19 Functional Status scale

45. LC. 2 How much are you currently affected in your everyday life by COVID-19? (Please indicate which one of the following statements applies to you most)

Mark only one oval.

- I have no limitations in my everyday life and no symptoms, pain, depression or anxiety related to the infection.
- I have negligible limitations in my everyday life as I can perform all usual duties/activities, although I still have persistent symptoms, pain, depression or anxiety.
- I suffer from limitations in my everyday life as I occasionally need to avoid or reduce usual duties/activities or need to spread these over time due to symptoms, pain, depression or anxiety. I am, however, able to perform all activities without any assistance.
- I suffer from limitations in my everyday life as I am not able to perform all usual duties/ activities due to symptoms, pain, depression or anxiety. I am, however, able to take care of myself without any assistance.
- I suffer from severe limitations in my everyday life: I am not able to take care of myself and therefore I am dependent on nursing care and/or assistance from another person due to symptoms, pain, depression or anxiety

Section F3 - Long COVID Symptoms

46. LC. 3 After which instance of COVID infection did you experience these effects on your daily life as listed above?

Mark only one oval.

- The first one
- The second one
- The third one
- The fourth one
- The fifth one
- After the fifth one
- I don't remember
